# R_2_O_3_ (R = La, Y) modified erbium activated germanate glasses for mid-infrared 2.7 μm laser materials

**DOI:** 10.1038/srep13056

**Published:** 2015-08-17

**Authors:** Muzhi Cai, Beier Zhou, Fengchao Wang, Tao Wei, Ying Tian, Jiajia Zhou, Shiqing Xu, Junjie Zhang

**Affiliations:** 1 College of Materials Science and Engineering, China Jiliang University, Hangzhou, 310018, China

## Abstract

Er^3+^ activated germanate glasses modified by La_2_O_3_ and Y_2_O_3_ with good thermal stability were prepared. 2.7 μm fluorescence was observed and corresponding radiative properties were investigated. A detailed discussion of J–O parameters has been carried out based on absorption spectra and Judd–Ofelt theory. The peak emission cross sections of La_2_O_3_ and Y_2_O_3_ modified germanate glass are (14.3 ± 0.10) × 10^−21^ cm^2^ and (15.4 ± 0.10) × 10^−21^ cm^2^, respectively. Non-radiative relaxation rate constants and energy transfer coefficients of ^4^I_11/2_ and ^4^I_13/2_ levels have been obtained and discussed to understand the 2.7 μm fluorescence behavior. Moreover, the energy transfer processes of ^4^I_11/2_ and ^4^I_13/2_ level were quantitatively analyzed according to Dexter’s theory and Inokuti–Hirayama model. The theoretical calculations are in good agreement with the observed 2.7 μm fluorescence phenomena. Results demonstrate that the Y_2_O_3_ modified germanate glass, which possesses more excellent spectroscopic properties than La_2_O_3_ modified germanate glass, might be an attractive candidate for mid-infrared laser.

In recent decades erbium doped materials have become a research hotspot due to 2.7 μm mid-infrared emission, which has wide applications in both civilian and military fields such as remote sensing, atmosphere pollution monitoring, eye-safe laser radar, medical surgery and precision guidance^1^,^2^,^3^,^4^. Er^3+^ is an ideal luminescent center for 2.7 μm emission corresponding to the ^4^I_11/2_ → ^4^I_13/2_ transition. However, for developing more efficient optical devices, besides the active ions, the host material must be considered as well. Thus, seraching for suitable host materials for mid-infrared lasers operating in this wavelength is essential.

Glass continues to attract considerable research interest owing to its feasibility of fabrication and ability to be used as high power solid state laser hosts. The host glasses for mid-infrared emission are expected to possess a minor absorption coefficient in the typical OH^−^ absorption at ~3 μm, low nonradiative decay rates and high radiative emission rates^5^,^6^. Therefore, a lot of investigations have been focused on fluoride and chalcogenide glasses, especially fluoride glass, mainly owing to their low phonon energy and low OH^−^ content. Although chalcogenide glass has quite low phonon energy and larger refractive index, its preparation process is fairly complex^7^. Compared to chalcogenide glass, fluoride glass is easily prepared and possesses low phonon energy, high rare earth solubility and extremely low OH^−^ content^8^,^9^. Moreover, the fluorozirconate system (ZBLAN) has been made into fiber laser in 1999 and its output power can reach 1.7 W^2^. Nevertheless, the low mechanical strength and damage threshold limit its further applications in high power or energy fiber laser systems.

The thermal stability, chemical durability, mechanical strength of germanate glass are superior to fluoride glass^8^. Besides, germanate glass also possesses higher solubility of rare earth ions than chalcogenide glass^7^. Moreover, germanate glass has the advantages of low phonon energy and good infrared transmission in a wide wavelength range compared with other oxide glass^10^,^11^. Thus, from the viewpoint of technological application, germanate glass is quite suitable as host material for mid-infrared laser^12^. Particularly, barium gallo-germanate (BGG) glass has been investigated extensively as an exist window for high energy lasers operating in the infrared wavelength region^13^,^14^. However, BGG glass has the disadvantages of high melting temperature, high viscosity and a large number of hydroxyl groups^15^,^16^. A high concentration of hydroxyl groups might lead to a strong absorption band around 2.7 μm and it is harmful for corresponding mid-infrared emission. To address these questions, some fluorine could be added into germanate glass since fluoride ions (F^−^) proved to be capable of decreasing melt viscosity and minimizing the OH^−^ content^15^,^16^,^17^. Additionally, germanate glass can be modified by adding or substituting other components such as La_2_O_3_ and Y_2_O_3_. The addition of La_2_O_3_ is expected to improve glass forming ability, while Y_2_O_3_ component is expected to improve thermal stability and further reduce OH^−^ content in glass due to its collection of non-bridging oxygen of glass^18^.

Although the difference of thermal and physical properties of La_2_O_3_ and Y_2_O_3_ modified germanate glass has been investigated by Jewell *et al.*^19^, there are few reports on the spectroscopic properties and mid-infrared emissions of La_2_O_3_ and Y_2_O_3_ modified germanate glass. In this paper, the thermal stability and spectroscopic properties of GeO_2_-Ga_2_O_3_-BaO-R_2_O_3_-5NaF-Er_2_O_3_ system (R = La, Y) under the excitations of 808 nm LD were investigated. The research of 2.7 μm spectroscopic properties and corresponding energy transfer mechanism in both La_2_O_3_ and Y_2_O_3_ modified germanate glasses has been carried out. Besides, energy transfer microscopic parameters were calculated via Dexter’s theory and Inokuti–Hirayama model for better understanding of energy transfer processes of Er^3+^ ions.

**Experimental processes.** Er^3+^ doped germanate glasses were synthesized by conventional melting method, which has the following molar compositions: 65GeO_2_-15Ga_2_O_3_-5BaO-(10-x)La_2_O_3_-x

Y_2_O_3_-5NaF-0.5Er_2_O_3_, (x = 0, 10), denoted as GL, GY, respectively. Samples were synthesized by using high-purity of GeO_2_, Ga_2_O_3_, BaO, La_2_O_3_, Y_2_O_3_, NaF and Er_2_O_3_ powders. The stoichiometric chemicals were well-mixed and melted at 1400 °C for 30 min in a covered alumina crucible. The melts were poured onto a preheated steel plate and pressed by another plate for shaping. After annealing at around glass transition temperature, all samples were cut and polished into 10 × 10 × 1.5 mm^3^ for further measurement.

Refractive indexes of samples were measured by prism minimum deviation method at the wavelength of 1053 nm. The resolution of the instrument was ±0.5 × 10^−4^. The densities were tested by Archimedes principle using distilled water as an immersion liquid with error limit of ±0.05%. Differential scanning calorimeter (DSC) curve is measured using NETZSCH DTA 404 PC at the heating rate of 10 K/min with maximum error of ±5 °C. Absorption spectra were recorded with a Perkin-Elmer- Lambda 900UV/VIS/NIR spectrophotometer in the range of 350-1640 nm. Photoluminescence spectra in the ranges of 2600–2800 nm and 1400–1700 nm were determined via a combined fluorescence lifetime and steady state spectrometer (FLSP 920) (Edingburg Co., England), which was detected with a liquid-nitrogen-cooled PbS detector using an 808 nm laser diode (LD) as an excitation source. The 808 nm LD with the same power was also utilized to measure the lifetimes of Er^3+^:^4^I_11/2_ and ^4^I_13/2_ levels. The lifetimes were calculated by fitting a single exponential function to the measured data. The same experimental conditions for different samples were maintained so as to get comparable results. All the measurements were performed at ambient temperature.

## Results and Discussion

### Thermal stability and density

**Figure 1:**
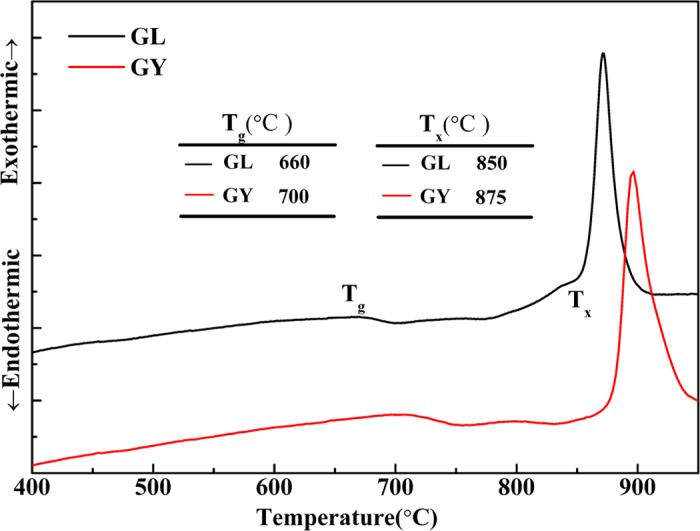
DSC curve of the prepared samples.

Figure 1 shows the differential DSC curves for the prepared glasses. The glass transition temperature T_g_, onset crystallization temperature T_x_, and thermal stability ΔT( = T_x_–T_g_) in various glasses are displayed in Table 1. It is found that the ΔT of GL and GY samples are 190 °C and 175 °C, respectively. Compared with ∆T, the glass formation factor, K_gl_ = (T_x_-T_g_)/(T_m_-T_g_), where T_m_ is the glass melting temperature, is more suitable to estimate the glass thermal stability. It is clear that K_gl_ of GL and GY can reach 0.26 and 0.25, respectively. Both ΔT and K_gl_ of prepared samples are larger than those of tellurite^20^, bismuth^21^ and germanate glass^22^, while is comparable to BGG glass^19^. The result suggests that the prepared germanate glasses have good glass forming ability and thermal stability.

T_g_ is an important factor for laser glass, which gives glass good thermal stability to resist thermal damage at high pumping intensities. The values of T_g_ of both prepared samples are substantially larger than the other glasses in Table 1. It is interesting to find that the T_g_ of GY is larger than GL, which is in accordance with the work of John M. Jewell^19^. The importance of the density for describing the structure of a glass is evident. The density of glass is mainly influenced by the molecular weight of glass components, the integration and the compactness of the glass network. The density of GL (4.76) is larger than GY (4.47), and a most possible reason for the decrement in density is ascribed to the smaller molecular weight of Y_2_O_3_ compared to La_2_O_3_. Both of the prepared glasses have a smaller density than BGG glass (4.85)^13^.

### Absorption spectra and J-O analysis

**Figure 2:**
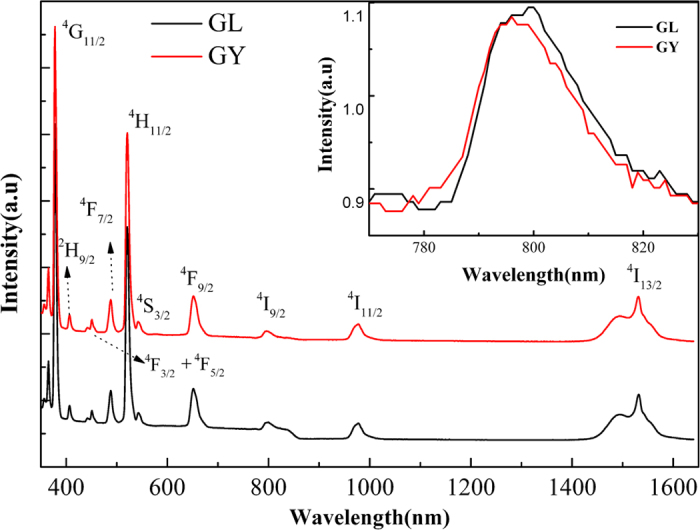
Absorption spectrum of the prepared samples. The inset is the enlarged absorption spectrum from 770–830 nm.

Figure 2 reveals the absorption spectra of Er^3+^ activated germanate glasses modified with La_2_O_3_ (GL) and Y_2_O_3_ (GY) at room temperature in wavelength region of 380–1600 nm. Absorption bands in this figure are labeled, which correspond to the transitions starting from the ^4^I_15/2_ ground state to higher ^4^I_13/2_, ^4^I_11/2_, ^4^I_9/2_, ^4^F_9/2_, ^4^S_3/2_, ^2^H_11/2_, ^4^F_7/2_, ^4^F_5/2,3/2_, ^2^H_9/2_ and ^4^G_11/2_ levels^23^. The shape and peak positions of each transition in present glasses are very similar to those of other Er^3+^ doped glasses^24^,^25^, except for some tiny divergences that originated from the different ligand field strength of host glasses. It is observed that two absorption peaks (^4^G_11/2_ → ^4^I_15/2_ and ^2^H_11/2_ → ^4^I_15/2_) are much stronger than other bands. They are sensitive to small changes of local environment around Er^3+^ ions, called hypersensitive transitions (HSTs)^5^. The inset of Fig. 2 displays the enlarged absorption spectrum in the range of 770–830 nm. Obvious absorption peaks around 808 nm manifested the prepared glasses can be pumped by low-cost 808 nm laser diodes (LDs).

Some important spectroscopic and laser parameters of rare earth doped glasses have been commonly analyzed by way of Judd-Ofelt (J-O) theory based on absorption data^26^,^27^,^28^. Details of the theory and method have been well described elsewhere^29^,^30^,^31^. Thus, only results will be presented here. The J-O intensity parameters Ω_t_ (t = 2, 4, 6) of GL and GY glass were determined in Table 1. The root-mean-square deviation (δ_rms_) in GL and GY glass is as low as 0.49 × 10^−6^, 0.29 × 10^−6^, respectively, proving the validity of the results and the reliable calculations.

It can be seen from Table 2 that the value of Ω_2_ in GY glass is higher than those of other Er^3+^ doped glasses. It is well known that Ω_2_ is strongly dependent on the RE^3+^ local environment and it is directly related to the symmetry or polarization of local structure and the covalence of chemical bonds formed by the RE^3+^ with its ligands. Based on this idea, the higher Ω_2_ in GY glass indicates the larger polarization of Y_2_O_3_ and asymmetry around Er^3+^ 32. Thus, the chemical bonds associated with the Er^3+^ ions is more covalent than those of silicate^33^, tellurite^34^, fluoride^6^ glasses as shown in Table 2. In addition, in this work, the Ω_2_ value of GL glass is lower than that of GY glass. According to the electronegativity theory, the covalency of the bond will become stronger with the decrease of the difference of electronegativity between cation and anion^35^. Since the values of electronegativity, for La, Y and O elements, are 1.1, 1.22, 3.5, respectively, the covalency of Y-O bond is stronger than that of La-O bond. This behavior will lead to the larger polarization of Y_2_O_3_ component than that of La_2_O_3_ and the asymmetry of the site occupied by Er^3+^ in GL glass is lower than that of GY glass. On the other hand, the Ω_6_ parameter is related to the overlap integrals of 4f and 5d orbit^36^. The Ω_6_ of GY is larger than those of GL, germanate^37^, tellurite^34^, silicate^33^ glasses, smaller than that of fluoride glass^6^.

### Radiative properties

Since S_md_ is independent of ligand fields and S_ed_ is a function of glass structure and composition^38^, in order to get flat emission spectrum, it can be effective to increase the relative contribution of the electric-dipole transition^39^. According to Judd-Ofelt theory, the line strength of the electric dipole components of 2.7 um emissions can be expressed as


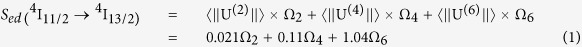


According to Eq. (1), the S_ed_ is mainly dominated by Ω_6_. From Table 2 it is noted that Ω_6_ in GY glass is higher than those of other various glasses except fluoride glass. Therefore, compared to GL glass, GY glass is more expected to be an appropriate host material that gets flat emission spectrum from the Er^3+^: ^4^I_11/2_ → ^4^I_13/2_ transitions.

Further calculation about spontaneous radiative transition probability (A_rad_), fluorescence lifetime (τ_rad_), and branching ratios (β) of Er^3+^ various transition in prepared glasses are listed in Table 3. As is shown in Table 3, the GY glass possesses a larger A_rad_ (36.21 s^−1^) than that of GL glass (35.03 s^−1^) for Er^3+^:^4^I_11/2_ → ^4^I_13/2_ respectively^40^. It is worth noting that both of prepared glasses possess evidently larger A_rad_ than BGG glass (19 s^−1^)^41^. Furthermore, the values of β in both samples are comparable to those germanate glasses^37^,^41^.

Since multiphonon relaxation rate has a substantial impact on 2.7 μm emissions, a low nonradiative decay rate is required to achieve strong 2.7 μm fluorescence. The multiphonon relaxation rate constant (k_mp_) from a given excited state can be estimated from the energy-gap law^42^. The multiphonon relaxation rate constant (k_mp_) can be defined as,


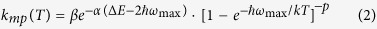


where *ΔE* is the energy gap between the emitting level and the adjacent lower level. *α* and *β* are positive-definite constants depending on glasses. *ћω*_*max*_ is the highest phonon energy of the glass. Where 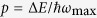 is the minimum number of phonons required to bridge the energy gap ΔE^43^. In this work, k_mp_ is calculated using the parameters α = 4.6 × 10^−3^ cm, β = 6.1 × 10^7^ s^−1^ and T = 300 K reported for germanate glass^44^. Via Eq. (2), the k_mp_ values for Er^3+^:^4^I_11/2_ → ^4^I_13/2_ and ^4^I_9/2_ → ^4^I_11/2_ transitions of GL glass and GY glass are determined as shown in Table 3. The transition of ^4^I_9/2_ → ^4^I_11/2_ is proposed to be a multiphonon decay process compared to ^4^I_11/2_ → ^4^I_13/2_ transition. In addition, the k_mp_ of ^4^I_11/2_ → ^4^I_13/2_ transition in both GL glass and GY glass are smaller than those of silicate glass (7.13 × 10^4^ s^−1^)^45^ and other germanate glass (1.11 × 10^4^ s^−1^)^45^, while comparable to fluorphosphate glass^45^.

### Fluorescence spectra at 2.7 μm

**Figure 3:**
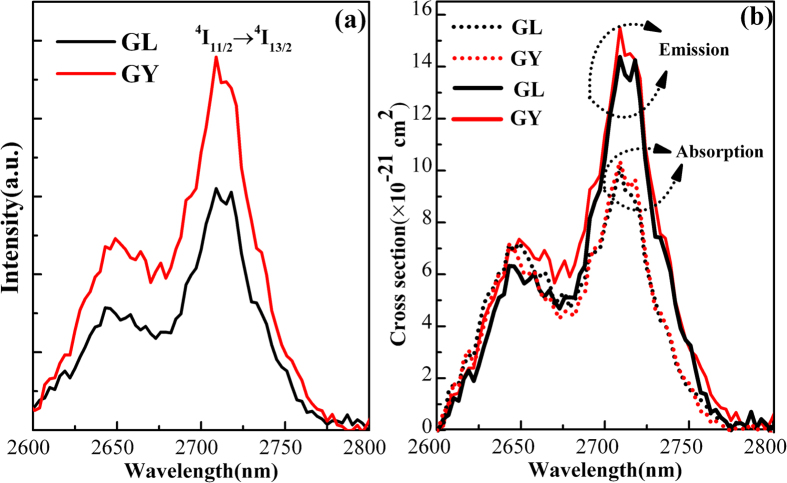
The fluorescence spectrum of the prepared samples at 2.7um (**a**) and the cross sections (emission and absorption) of prepared samples (**b**).

Figure 3 illustrates the mid-infraed emission spectra and cross sections of Er^3+^ doped GL and GY glasses pumped at 808 nm laser diode(LD). As shown in Fig. 3(a), the 2.7 μm emissions of both glasses can be observed. It is interesting to find that the 2.7 μm emission intensity of GY glass is stronger than GL glass. Some spectroscopic parameters have been calculated to better explain the interesting situation.

According to the Fuchtbauer-Ladenburg theory the 2.7 μm emission cross section (σ_em_) can be calculated by


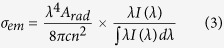


where λ is the emission wavelength, A_rad_ is the spontaneous radiative transition probability of Er^3+^: ^4^I_11/2_ → ^4^I_13/2_ transition, c is the velocity of light in vacuum, n is the refractive index of glass host (GL:1.76 and GY: 1.73), I(λ) is the 2.7 μm fluorescence intensity, and ∫I(λ)dλ is the integrated fluorescence intensity. Based on emission cross section, σ_em_, absorption cross section (σ_abs_) can be obtained by[51],


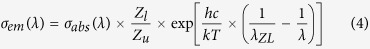


where Z_l_ and Z_u_ are the partition functions for the lower and the upper levels involved in the considered optical transition, respectively. *T* is the temperature (here is 300 K), *k* is the Boltzmann constant and *λ*_*ZL*_ is the wavelength for the transition between the lower Stark sublevels of the emitting multiplets and the lower Stark sublevels of the receiving multiplets.

As shown in Fig. 3(b), the absorption and emission cross sections can be calculated by Eq. (3) and (4). It can be seen that the peak absorption cross sections at 2.7 μm of GL and GY glass are (10.3 ± 0.10) × 10^−21^ cm^2^ and (10.1 ± 0.10) × 10^−21^ cm^2^, respectively, the peak emission cross section are (14.3 ± 0.10) × 10^−21^ cm^2^ and (15.4 ± 0.10) × 10^−21^ cm^2^, respectively. Higher emission cross section means that better laser gain can be achieved in glass. It is found that the obtained σ_em_ for both glasses are higher than those of fluoride (9.16 × 10^−21^ cm^2^)^6^, bismuthate (7.73 × 10^−21^ cm^2^)^21^ and tungsten- tellurite glass (6.05 × 10^−21^ cm^2^)^46^.

In addition, according to Eq. (5), the effective emission bandwidths (Δλ_eff_) have been obtained.


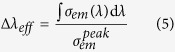


where 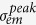 is the peak emission cross section at 2.7 μm. Since the 2.7 μm emission band of Er^3+^ ions in glass is asymmetric, it is more reasonable to select effective emission bandwidth other than the full width at half maximum as presented in Fig. 3(b). For broadband amplifier, it is required that effective emission bandwidth is as wide as possible to provide multiple channels for signal transmission. It is calculated from Fig. 3(a) that the *Δλ*_*eff*_ of GL and GY glass can reach 58.4 and 59.3 nm, which is larger than that of chalcogenide glass (56 nm)^5^. High effective emission bandwidth means that the prepared glasses have potential applications in broadband amplifier operating at 2.7 μm.

**Figure 4:**
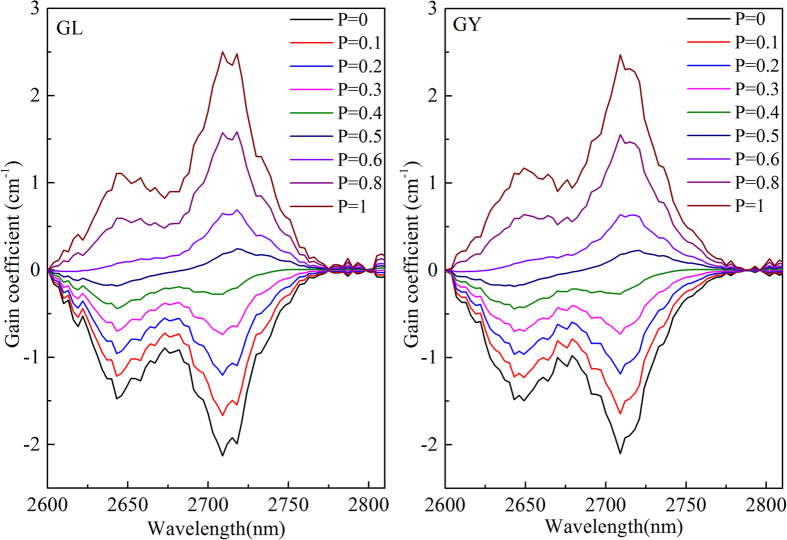
Gain spectra of GL (left) and GY (right) glasses.

According to the σ_em_(λ) and σ_abs_(λ), the gain spectra (G(λ)) at 2.7 μm can be calculated by the report^18^. Figure 4 indicates the gain spectra of 2.7 μm of prepared glasses. Evidently, both GL and GY, when the population inversion P > 0.4, the gain cross sections in range of 2683-2772 nm become positive. It is suggested that Er^3+^ activated GL and GY glass is an attractive candidate for mid-infrared laser with low pump threshold.

The product of 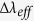 × 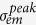, defined as gain bandwidth, is another important parameter to evaluate the gain performances of prepared samples^47^. Larger gain bandwidth means better gain property of the material. Due to higher emission cross section and larger emission bandwidth, the GY glass has higher gain performance (9.13 × 10^−26^ cm^3^) than GL glass (8.35 × 10^−26^ cm^3^). From the above comparsion, it is roundly expected that GY glass have a better gain properties than GL glass at 2.7 μm, which is also in good with the 2.7 fluorescence intensity as shown in Fig. 3(a).

### Energy transfer mechanism and microparameters

**Figure 5:**
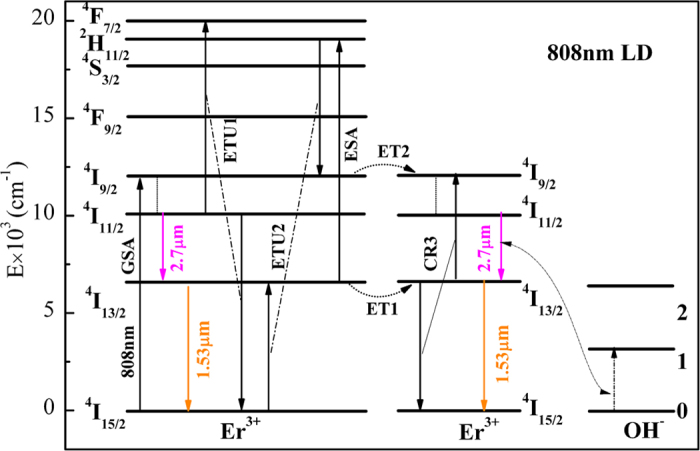
The energy transfer mechanism of GL and GY.

Figure 5 reveals the energy transfer process of Er^3+^ pumped by 808 nm LD. Under 808 nm pumping, the ions in Er^3+^: ^4^I_15/2_ level are excited to the ^4^I_9/2_ state by ground state absorption process (GSA). Then the ions in ^4^I_9/2_ level non-radiatively decay to ^4^I_11/2_ state by multiphonon relaxation process due to small energy gap between ^4^I_9/2_ and ^4^I_11/2_ level. The ions in ^4^I_11/2_ state are populated to ^4^F_7/2_ level owing to excited state absorption (ESA: ^4^I_11/2_ + a photon → ^4^F_7/2_) or energy transfer upconversion (ETU1: ^4^I_11/2_ + ^4^I_11/2_ → ^4^I_15/2_ + ^4^F_7/2_). Afterwards, ions in ^4^F_7/2_ level relax non-radiatively to ^2^H_11/2_ state due to multiphonon relaxation process. Due to small energy gaps among ^2^H_11/2_, ^4^S_3/2_ and ^4^F_9/2_ levels, ions in ^2^H_11/2_ state decay nonradiatively to ^4^S_3/2_ and ^4^F_9/2_ level. On the other hand, ions in ^4^I_11/2_ level can decay to lower ^4^I_13/2_ level by radiative or nonradiative process and radiative process generates 2.7 μm fluorescence. Finally, ions in ^4^I_13/2_ state relax radiatively to the ground state and 1.53 μm fluorescence occurs. Besides, ions in ^4^I_13/2_ level can also undergo ETU2 process (^4^I_13/2_ + ^4^I_13/2_ → ^4^I_15/2_ + ^4^I_9/2_), thus resulting in the further population accumulations of ^4^I_9/2_ level. The nonradiative rate of ^4^I_9/2_ → ^4^I_11/2_ transition is so large that ions in ^4^I_9/2_ level decay quickly to ^4^I_11/2_ level, which is beneficial to populations of the ^4^I_11/2_ level and 2.7 μm emissions. Moreover, as is discussed in above, for Er^3+^: ^4^I_11/2_ → ^4^I_15/2_ transition, GY has a higher multiphonon relaxation rate constant than GL, which makes it have a more active energy transfer process mentioned above. It is worth mentioning that the residual OH^−^ of glass is to the disadvantage of mid-infrared emission. It can quench the 2.7 μm fluorescence via the following processes (^4^I_11/2_ + 0 → ^4^I_13/2_ + 1) in prepared glasses as depicted in Fig. 5. These energy transfer processes are harmful for mid-infrared emissions. Hence, it is necessary to minimize OH^−^ content and weaken unwanted energy transfer from Er^3+^ to OH^−^.

To make clear of mid-infrared emission mechanism, a quantitative understanding of energy transfer process about Er^3+^: ^4^I_11/2_ level in present glasses is required. According to FÖster^48^ and Dexter^49^, the probability rate of energy transfer between donor and acceptor can be estimated as^50^,^51^


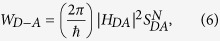


Where 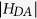 is the matrix element of the perturbation Hamiltonian between initial and final states in energy transfer process, *N* is the total phonons in the transfer process *m* + *k* = *N*, 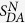 is the integral overlap between the m-phonon emission sideband of donor ions and k-phonon absorption line shapes of acceptor ions. In our case, both donor and acceptor are Er^3+^ ions on ^4^I_11/2_ level. In the case of weak electron-phonon coupling, it is suitable for rare earth ions. 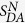 can be approximated by


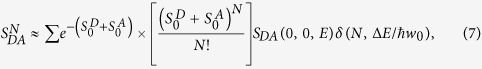


Where 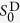 and 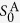 are the Huang-Rhys factors, *S*_*DA*_ (0, 0, *E*) is the overlap between the zero phonon line shape of emission and absorption. Then the integral overlap in the case of m-phonon emission by the donor and no phonon involvement by the acceptor can be expressed as


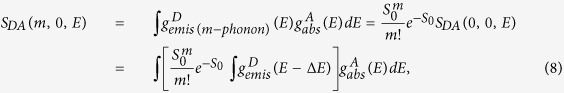


Where *ΔE* = *mhω*_*0*_. Since the measurements are carried out at some finite temperature T, the multi-phonon probability must be included, and the emission cross section (*σ*_*emis*_) with m phonon emission and absorption cross section (*σ*_*abs*_) with k phonon absorption can be proposed as


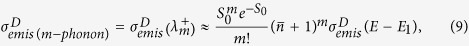



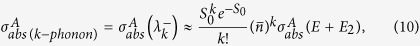


Where *E*_*1*_* = mhω*_*0*_, *E*_*2*_* = khω*_*0*_, and *ΔE* = E_1_ + E_2_, 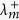
denotes the transiation of emission cross section wavelength by m-phonon emission, 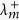 = *1/(1/λ- mhω*_*0*_), and 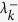 represents the translation of absorption cross section spectra wavelength due to k-phonon absorption 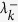 = *1/(1/λ + mhω*_*0*_).

The energy transfer coefficient is then expressed by


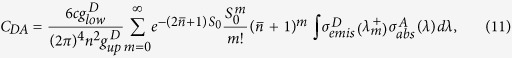


In this work, both the donor and acceptor are Er^3+^ ions. Energy transfer properties of ^4^I_11/2_ and ^4^I_13/2_ level in GL and GY have been calculated using Eqs (6)–(11), 14, 17, 18, 19, 24 and listed in Table 4. The results show that the energy transfer processes of Er^3+^: ^4^I_11/2_ and ^4^I_13/2_ level are scarcely phonon dependent. The energy transfer coefficient C_D-A_ in GL and GY of Er^3+^: ^4^I_13/2_ level are as high as 50 × 10^−40^ and 52 × 10^−40^ cm^6^/s, respectively, but ^4^I_11/2_ level is 4.61 × 10^−40^ and 4.63 × 10^−40^ cm^6^/s, respectively. This suggests the energy of ^4^I_13/2_ level in present glasses can more efficiently transfer to the same level nearby compared with ^4^I_11/2_ level, which is helpful to deplete the populations of ^4^I_13/2_ level and promote population inversion between ^4^I_11/2_ and ^4^I_13/2_ level.

**Figure 6:**
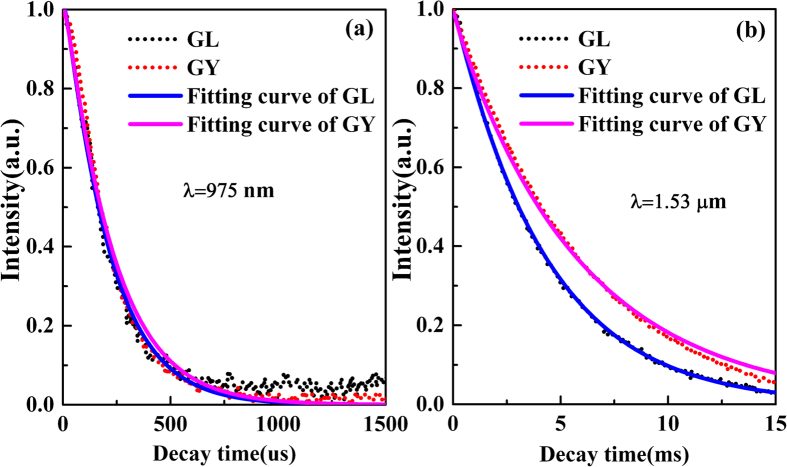
Decay data (dash line) of ^4^I_11/2_ level at 975 nm (**a**) and I_13/2_ level at 1530 nm (**b**) in prepared glasses together with fitting curves (solid line) via I-H model.

Figure 6 displays the decay curves of ^4^I_11/2_ and ^4^I_13/2_ level in both glasses pumped by 808 LD. It is found that the decay tendency of GY is slower than GL at both 975 and 1530 nm. To shed new light on the population behavior of ^4^I_11/2_ level and ^4^I_13/2_ level, the energy transfer processes of these energy levels were analyzed quantitatively on the basis of Inokuti–Hirayama (I-H) model. I-H model can also be used to estimate the energy transfer process among Er^3+^ ions, which is expressed as^52^,^53^


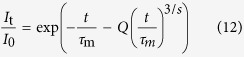


where s is 6, 8 or 10 depending on whether the dominant mechanism of interaction is dipole-dipole, dipole-quadrupole or quadrupole-quadrupole, respectively. τ_0_ is the intrinsic lifetime. The energy transfer parameter (Q) is defined as


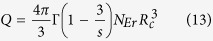


where Γ(1-3/s) is equal to 1.77 for dipole-dipole interactions (s = 6), 1.43 for dipole- quadrupole interactions (s = 8) and 1.3 in the case of quadrupole-quadrupole interactions (s = 10). N_Er_ is the concentration of Er^3+^ ions (in ions cm^−3^) and R_c_ is the critical transfer distance defined as the donor-acceptor separation for which the energy transfer rate is equal to the rate of intrinsic decay of the donors.

The decay curves of present samples have been well fitted by I-H model for s = 6 and the results are listed in Table 5. This indicates that the energy transfer among Er^3+^ ions takes place due to dipole-dipole interactions. From Table 5, it can be found that the energy transfer parameter Q for GY sample is lower than that of GL sample in ^4^I_11/2_ level while the Q value of GY sample is higher than that of GL sample in ^4^I_13/2_ level. The higher the value Q is, the stronger the energy transfer process becomes. It is indicated that higher Q of ^4^I_13/2_ level and lower Q of ^4^I_11/2_ level for GY sample are more beneficial for the population inversion between them and enhancing 2.7 μm emissions. It is in accordance with the result of Fig. 3(a).

## Conclusions

Er^3+^ doped germanate glasses modified by La_2_O_3_ and Y_2_O_3_ with good thermal stability were prepared. 2.7 μm fluorescence was observed and corresponding radiative properties were investigated. J–O parameters have been discussed in detail based on absorption spectra and Judd–Ofelt theory. The peak emission cross sections of La_2_O_3_ and Y_2_O_3_ modified germanate glass are (14.3 ± 0.10) × 10^−21^ cm^2^ and (15.4 ± 0.10) × 10^−21^ cm^2^, respectively. To understand the 2.7 μm fluorescence, non-radiative relaxation rate constants and energy transfer coefficients of ^4^I_11/2_ and ^4^I_13/2_ levels have been obtained and discussed. Moreover, the energy transfer processes of ^4^I_11/2_ and ^4^I_13/2_ level were quantitatively analyzed according to Dexter’s theory and Inokuti–Hirayama model. The theoretical calculations are in good agreement with the observed 2.7 μm fluorescence phenomena. Results demonstrate that the Y_2_O_3_ modified germanate glass possesses more excellent spectroscopic properties than La_2_O_3_ modified germanate glass, and might be an attractive candidate for mid-infrared laser materials.

## Additional Information

**How to cite this article**: Cai, M. *et al.* R_2_O_3_ (R =La, Y) modified erbium activated germanate glasses for mid-infrared 2.7 µm laser materials. *Sci. Rep.* **5**, 13056; doi: 10.1038/srep13056 (2015).

## Figures and Tables

**Table 1:** The measured glass transition temperature (T_g_), the onset crystallization temperature (T_x_) and the calculated thermal stability (ΔT) in various glasses.

**Sample**	**T**_**g**_ **(°C)**	**T**_**x**_ **(°C)**	**ΔT (°C)**	**K**_**gl**_	**Reference**
GL	660	850	190	0.26	This work
GY	700	875	175	0.25	This work
Tellurite	354	498	146	0.19	20
bismuth	365	511	146	0.24	21
germanate	618	747	123	0.176	22

**Table 2:** J-O intensity parameters Ω_t_ (t = 2,4,6) (×10^−20^ cm^2^) of Er^3+^ in various glasses.

**Samples**	**Ω**_**2**_	**Ω**_**4**_	**Ω**_**6**_	**δ**_**r.m.s**_ (×**10**^−**6**^)	**References**
GL	5.18 ± 0.06	2.36 ± 0.04	0.78 ± 0.07	0.49	This work
GY	5.72 ± 0.03	2.02 ± 0.05	1.04 ± 0.03	0.29	This work
silicate	4.23	1.04	0.61	5.4	33
Tellurite	3.40	1.00	0.20	–	34
Fluoride	3.08	1.46	1.69	0.32	6

**Table 3:** Experimental (*f*_exp_) and calculated (*f*_cal_) oscillator strengths for selected transitions of Er^3+^ in various glasses.

**Transitions**	**ΔE (cm**^**−1**^)	**GL**			**GY**		
		**A**_**rad**_**(s**^−**1**^)	**β (%)**	**τ**_**rad**_**(ms)**	**A**_**rad**_**(s**^−**1**^)	**β (%)**	**τ**_**rad**_**(ms)**
^4^I_13/2_ → ^4^I_15/2_	6532	170.20	100	5.88	184.57	100	5.42
^4^I_11/2_ → ^4^I_15/2_	10225	150.70	81.14	5.38	176.28	82.96	4.71
→^4^I_13/2_	3693	35.03	18.86	–	36.21	17.04	–
^4^I_9/2_ → ^4^I_15/2_	12500	253.86	85.01	3.35	205.51	79.14	3.85
→^4^I_13/2_	5968	41.41	13.87	–	50.99	19.64	–
→^4^I_11/2_	2275	3.34	1.12	–	3.19	1.23	–
^4^F_9/2_ → ^4^I_15/2_	15337	2289.43	92.04	0.40	2065.61	91.27	0.44
→^4^I_13/2_	8806	125.63	5.05	–	111.82	4.94	–
→^4^I_11/2_	5112	67.79	2.73	–	81.10	3.58	–
→^4^I_9/2_	2837	4.61	0.19	–	4.69	0.21	–
^4^S_3/2_ → ^4^I_15/2_	18416	1040.44	66.07	0.64	1285.13	66.88	0.52
→^4^I_13/2_	11884	426.43	27.08	–	522.51	27.19	–
→^4^I_11/2_	8191	35.96	2.28	–	41.89	2.18	–
→^4^I_9/2_	5916	71.94	4.57	–	72.15	3.75	–
^2^H_11/2_ → ^4^I_15/2_	19194	10648.38	100	0.09	10224.71	100	0.10
^4^F_7/2_ → ^4^I_15/2_	20492	3359.68	99.24	0.30	3571.00	99.34	0.28
k_mp_	^4^I_11/2_ → ^4^I_13/2_	6.98 × 10^3^			6.6 × 10^3^		
(s^−1^)	^4^I_9/2_ → ^4^I_11/2_	4.75 × 10^6^			4.53 × 10^6^		

**Table 4:** Calculated energy transfer microscopic parameters C_DA_ for ^4^I_11/2_ and ^4^I_13/2_ levels in present glasses.

	**GL**			**GY**		
Transition	N (#phonons-assist) (% contribution)		C_DA_ (10^−40^ cm^6^·s^−1^)	N (#phonons-assist) (%contribution)		C_DA_ (10^−40^ cm^6^·s^−1^)
^4^I_11/2_ → I_11/2_	0 (99.99)	1 (0.01)	4.61	0 (99.99)	1 (0.01)	4.63
^4^I_13/2_ → I_13/2_	0 (99.99)	1 (0.01)	50	0 (99.99)	1 (0.01)	52

The number # of phonons necessary to assist the energy transfer process is also indicated with their contributions (in %).

**Table 5:** Lifetime (τ_0_), and energy transfer parameter (Q) of Er^3+^: ^4^I_11/2_ and ^4^I_13/2_ level in prepared samples.

**Sample**	**I–H model**			
	**975 nm**		**1530 nm**	
	**τ**_**m**_ **(ms)**	**Q**	**τ**_**m**_ **(ms)**	**Q**
GL	0.0018	0.23	4.17	0.043
GY	0.00197	0.21	6.11	0.053
